# Supplementary material for: Linking cortical surface area to computational properties in human visual perception
Source: iScience. 2024 Jul 22;27(8):110490. doi: 10.1016/j.isci.2024.110490 (PMC11325354; doi:10.1016/j.isci.2024.110490)
Supplement: Document S1. Data S1–S6 [file mmc1.pdf]

iScience, Volume *27*

Supplemental information

Linking cortical surface area to computational properties in human visual perception

Scott O. Murray, Tamar Kolodny, and Sara Jane Webb

Supplementary Information

Data S1: Normalized versus raw surface area, related to Figure 3.

Our hypothesis relating SA to circuitry was originally conceptualized as relating to the proportional enlargement or contraction of SA in a region, rather than the raw surface area of a region. We reasoned that input/output circuitry would simply scale with total brain size, so it was important to account for total surface area in the analyses. Normalizing SA had the added benefit of accounting for differences in total cortical SA that might exist between individuals or groups in our sample (e.g., males vs. females). However, we observed that for many of the relationships we report, normalizing SA may not be necessary. First, normalized (%) and raw (mm) SA are very strongly related (Fig. 9, $r_{60} = 0.76$, $p < 10^{-12}$). In addition, all of the identified parietal and frontal parcels identified using the correlation between normalized SA and thresholds were also significantly correlated when raw SA area was used. The fact that normalized and raw SA give similar results suggests that these areas contribute particularly strongly to overall cortical size—that is, if someone has a large cortex, these areas contribute particularly strongly to its size.

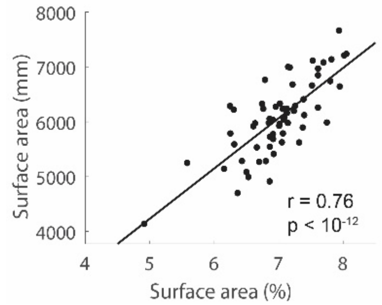

Supplemental Figure 1. Summed (across parietal and frontal regions) normalized SA (%) and raw SA (mm's) are highly correlated. Related to Figure 3.

Data S2: No evidence for relationships between visual area SA and behavior, related to Figure 3.

Previous research has demonstrated a link between larger regional SA and improved performance in tasks related to that brain region, suggesting a possible "more cortex is better" hypothesis connecting cortical SA and function. For example, increased SA in V1 is associated with better performance in acuity tasks[1,2]. Consequently, since thresholds are correlated with hMT+ responses[3,4], we might expect a correlation between SA in motion-sensitive regions like hMT+ and motion duration thresholds. Although our initial analyses did not reveal any significant correlations, it is important to note that extrastriate visual areas, like hMT+, are relatively small and exhibit low positional consistency across individuals, which could impact the strength of observed relationships. This anatomical variability might be responsible for the lack of strong correlations between regional SA in motion-selective regions and motion duration thresholds. Considering these factors, we conducted an exploratory analysis to investigate potential trend-level relationships.

First, we examined the individual correlation values between SA and thresholds in five regions known to exhibit motion-selective neural responses: V1, V3A, V6, MT, and MST. All correlations were non-significant ($p \geq 0.14$). Next, we explored a different cortical parcellation strategy that focused specifically on visually defined regions in the occipital, temporal, and parietal cortices ("Wang atlas"[5]). We replicated our basic finding using the new parcellation. Specifically, the SA of two right parietal regions of the Wang atlas showed significant negative correlations with duration threshold (right IPS3, $r_{55} = -0.34$, $p = 0.009$; right SPL, $r_{55} = -0.34$, $p = 0.01$). The SA of known visual motion processing regions such as MT and MST were not associated with duration thresholds (MT, $r_{55} = 0.21$, $p = 0.10$; MST, $r_{55} = 0.18$, $p = 0.20$). Overall, the results suggest that SA in visual motion processing regions, at least as commonly defined using atlas-based parcellations, are not associated with task performance.

Data S3: Consistent findings across alternative atlases (Desikan-Killiany), related to Figure 3.

Our primary analysis utilized the HCP-MMP atlas, which includes 360 regions, selected for its high degree of anatomical specificity and alignment with known functional boundaries. The extensive number of regions in this atlas, however, makes traditional multiple comparison corrections impractical. To mitigate potential issues of false positives, we adopted a multi-atlas strategy. This strategy assumes that replication of findings across different parcellation strategies—specifically the HCP-MMP, the Wang atlas for visual areas (see Supplemental Data 2, above), and the Desikan-Killiany (DK) atlas, which broadly categorizes the cortex—provides a robust check against false discoveries.

Using the same significance threshold of $p < 0.01$, only one region, the right superior temporal area, demonstrated a significant negative correlation with motion duration thresholds. This region encompasses many of the right parietal regions identified in the main HCP-MMP analysis. Additionally, the left inferior-frontal region (11L) identified in the HCP-MMP analysis corresponded with the left lateral orbitofrontal area in the DK atlas, which showed a negative correlation ($p = 0.04$). Overall, these findings suggest that the basic effect is robust across multiple parcellation methods.

Data S4: Left anterior temporal pole, related to Figure 3.

For completeness, we report an observed relationship between SA and duration thresholds in a region where we did not have strong *a priori* hypotheses: the left anterior temporal pole. This region is visible in Fig. 3A and includes parcels STGa, STSda, TE1a, and STSva. The relationship for any given parcel was modest, with *r*-values ranging from -0.27 to -0.29 and *p*-values from 0.03 to 0.05. The relationship between SA variation in the temporal pole did not extend to the alternate stimulus configuration (Experiment 2), the additional cohort of participants (Experiment 3), or the fMRI responses (Experiment 4). Therefore, we do not discuss its potential involvement in more detail other than to note it as a potential region of interest for future studies.

Data S5: Parameter Values and Their Differential Effects on Stimulus Size, related to Figure 4.

In this section, we present a more detailed analysis using a range of model parameter values to demonstrate the specific influence of different aspects of the divisive normalization model on visual motion perception. We specifically focus on how variations in the excitatory drive receptive field width (RF width) and top-down gain width (attention window) uniquely affect predicted duration thresholds for different stimulus sizes.

Our findings show that modifications to the excitatory RF width predominantly impact the predicted duration thresholds for small stimulus sizes (Supplemental Figure 2, left). Conversely, adjustments in top-down gain width are shown to differentially affect the thresholds for larger stimulus sizes. In the main text, we leverage this parameter-specific dissociation to infer the functional contributions of cortical surface area (SA) in the parietal and frontal regions.

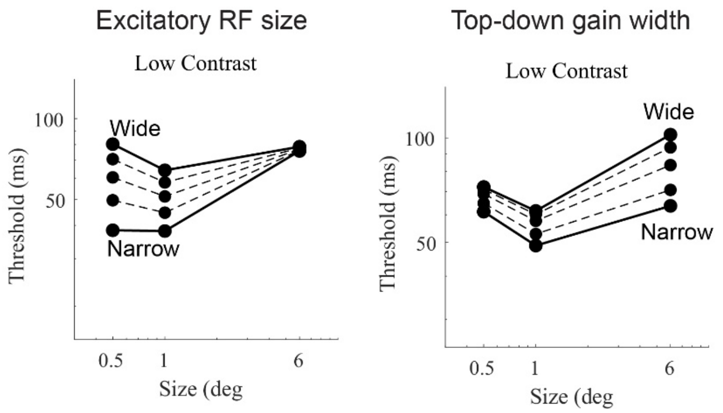

Supplemental Figure 2. Using a range of parameter values we show that excitatory RF size (left) predominantly affects predicted thresholds for small sizes and top-down gain width predominantly affects predicted thresholds for large sizes. Related to Figure 4.

Data S6: Psychophysical Data Quality, related to Figure 3.

Supplemental Figure 3 shows examples of different situations for four subjects: A) an excluded subject, B) a subject excluded after visual inspection (a case where the algorithm failed to detect several poor staircases), C) a subject included but with a single staircase removed from the final estimate, and D) a subject with all data included.

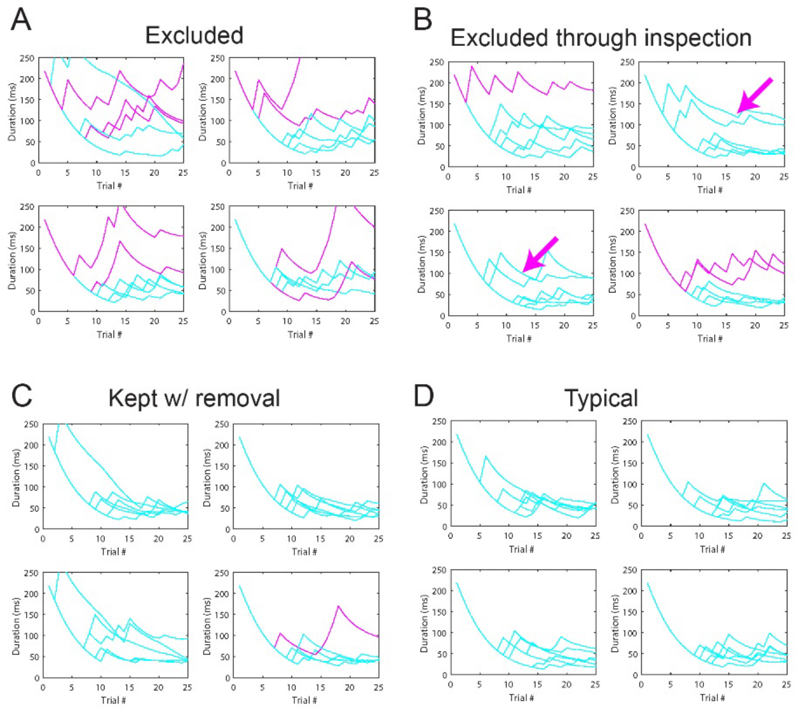

Supplemental Figure 3. Staircase examples. Each panel is a single run. Related to Figure 3. Each line is a staircase from a particular stimulus condition. (A) and (B) show examples of two of the eleven excluded subjects. Magenta lines are staircases highlighted by the data quality algorithm. (B) Subject was excluded after further visual inspection, noting the 4 staircases with early errors in the trials that prevented convergence along with the 3 magenta, flagged staircases. (C) and (D) are two examples of included subjects. (C) An example of a single staircase (magenta line) that was eliminated from the threshold estimates of an included subject. (D) A typical staircase pattern for a subject with all staircases included.

Supplemental References

1. Duncan, R.O., and Boynton, G.M. (2003). Cortical magnification within human primary visual cortex correlates with acuity thresholds. Neuron *38*, 659-671.
2. Song, C., Schwarzkopf, D.S., Kanai, R., and Rees, G. (2015). Neural population tuning links visual cortical anatomy to human visual perception. Neuron *85*, 641-656.
3. Murray, S.O., Schallmo, M.-P., Kolodny, T., Millin, R., Kale, A., Thomas, P., Rammsayer, T.H., Troche, S.J., Bernier, R.A., and Tadin, D. (2018). Sex differences in visual motion processing. Current Biology *28*, 2794-2799. e2793.
4. Schallmo, M.-P., Millin, R., Kale, A.M., Kolodny, T., Edden, R.A., Bernier, R.A., and Murray, S.O. (2019). Glutamatergic facilitation of neural responses in MT enhances motion perception in humans. NeuroImage *184*, 925-931.
5. Wang, L., Mruczek, R.E., Arcaro, M.J., and Kastner, S. (2015). Probabilistic maps of visual topography in human cortex. Cerebral cortex *25*, 3911-3931.
